# Association Between Timing of Preoperative Magnetic Resonance Imaging (MRI) and Postoperative Outcomes After Lumbar Decompression: A Propensity-Matched Cohort Study

**DOI:** 10.7759/cureus.108613

**Published:** 2026-05-10

**Authors:** Katelyn A Robertson, Rejoice Spivey, Kaitlyn Blake, Amber Courtland, Teneshia Huggins, Mrinalini Deverapalli, Miriam Michael

**Affiliations:** 1 Department of Neurosurgery, Howard University College of Medicine, Howard University Hospital, Washington, DC, USA; 2 Department of Radiology, Howard University College of Medicine, Howard University Hospital, Washington, DC, USA; 3 Department of Internal Medicine, Howard University College of Medicine, Howard University Hospital, Washington, DC, USA; 4 Department of Emergency Medicine, Howard University College of Medicine, Howard University Hospital, Washington, DC, USA; 5 Department of Pediatric Neurosurgery, Howard University College of Medicine, Howard University Hospital, Washington, DC, USA; 6 Department of Internal Medicine, Howard University Hospital, Washington, DC, USA

**Keywords:** infection, lumbar decompression, mri timing, postoperative outcomes, spine surgery

## Abstract

Background: Magnetic resonance imaging (MRI) is routinely used in preoperative planning for lumbar decompression, yet the optimal timing of imaging prior to surgery remains unclear. Variability in MRI timing may reflect differences in care pathways and could influence postoperative outcomes.

Objective: This study aims to evaluate the association between the timing of preoperative MRI and postoperative outcomes following lumbar decompression.

Methods: A retrospective cohort study was conducted using the TriNetX Global Collaborative Network (TriNetX, LLC, Cambridge, MA). Adult patients undergoing lumbar decompression for degenerative lumbar spine pathology were stratified based on timing of preoperative MRI: 31-90 and <30 days prior to surgery. Propensity score matching (1:1) was performed to balance baseline characteristics. Outcomes assessed within one year included reoperation, postoperative infection, and neurologic complications.

Results: After matching, 37,245 patients per cohort were included. There were no significant differences in reoperation (1.7% vs. 1.8%, risk ratio (RR) = 0.94, p = 0.46) or neurologic complications (0.1% vs. 0.1%, RR = 0.89, p = 0.56). Patients undergoing MRI 31-90 days prior to surgery had significantly lower rates of postoperative infection compared with those imaged within 30 days (2.7% vs. 3.3%, RR = 0.81, p < 0.001).

Conclusion: Timing of preoperative MRI was not associated with reoperation or neurologic complications following lumbar decompression. However, an MRI performed 31-90 days prior to surgery was associated with lower postoperative infection rates, suggesting that the timing of imaging may reflect underlying care pathways rather than a causal relationship.

## Introduction

Lumbar decompression surgery is a commonly performed intervention for degenerative spinal conditions, including lumbar spinal stenosis, intervertebral disc herniation, radiculopathy, and spondylolisthesis. The prevalence of these conditions has increased in parallel with an aging population, contributing to a marked rise in lumbar spine surgical interventions over the past two decades [1]. Magnetic resonance imaging (MRI) remains the primary modality for preoperative evaluation, providing critical information for diagnosis, anatomical localization, and surgical planning [2]. Consequently, MRI is a standard component of the preoperative workup for patients undergoing lumbar decompression.

Despite its widespread use, the optimal timing of preoperative MRI relative to surgical intervention remains unclear. In clinical practice, the interval between imaging and surgery varies considerably, ranging from studies obtained shortly before surgery to those performed several months prior. This variability may reflect differences in healthcare access, symptom progression, surgeon preference, and institutional logistics [3]. In some cases, repeat imaging is obtained when delays are prolonged due to concerns regarding the interval progression of pathology. However, the clinical significance of MRI timing on postoperative outcomes remains poorly defined.

Prior studies have largely focused on the diagnostic utility and cost considerations of spinal imaging, with limited evaluation of the temporal relationship between imaging and surgical intervention [4]. Importantly, shorter MRI-to-surgery intervals may reflect more acute or severe clinical presentations, whereas longer intervals may reflect elective care pathways, introducing confounding by indication rather than a direct effect of imaging timing itself [5]. As such, any observed associations between MRI timing and postoperative outcomes must be interpreted cautiously within the context of underlying clinical and care-delivery differences.

The objective of this study was to evaluate the association between the timing of preoperative MRI (≤30 vs. 31-90 days before surgery) and postoperative outcomes in patients undergoing lumbar decompression. We hypothesized that MRI timing would not be independently associated with major postoperative complications, and that any observed differences would more likely reflect underlying patient acuity and care pathways rather than a causal effect of imaging timing.

## Materials and methods

Study design and data source

This retrospective cohort study was conducted using the TriNetX Global Collaborative Network (TriNetX, LLC, Cambridge, MA), a federated health research platform that aggregates deidentified electronic health record data from multiple healthcare organizations [6]. The network includes data from approximately 171 institutions and captures longitudinal information on patient demographics, diagnoses, procedures, medications, and clinical outcomes [6].

The TriNetX platform enables real-time analysis of large-scale clinical data while maintaining patient privacy through deidentification in compliance with the Health Insurance Portability and Accountability Act. As all data are deidentified, this study was determined to be exempt from institutional review board approval.

All queries and analyses were conducted directly within the TriNetX platform using built-in analytical tools. Cohort queries and analyses were performed within the TriNetX environment, and full reproducibility may be limited due to the proprietary structure of the database. The availability of longitudinal patient records allows for the assessment of outcomes over specified follow-up periods. For this study, the Global Collaborative Network was used to identify patients undergoing lumbar decompression and to evaluate postoperative outcomes based on the timing of preoperative MRI.

Cohort selection

Adult patients (≥18 years) undergoing lumbar decompression for degenerative lumbar spine pathology were identified within the TriNetX Global Collaborative Network. Patients were required to have at least one diagnosis of degenerative lumbar spine disease, defined by the following International Classification of Diseases, 10th Revision (ICD-10) codes: lumbar spinal stenosis (M48.06), intervertebral disc displacement of the lumbar region (M51.26), lumbar radiculopathy (M54.16), or lumbar spondylolisthesis (M43.16).

Eligible patients were further required to have undergone lumbar decompression surgery, identified using the following Current Procedural Terminology (CPT) codes: 63030 (lumbar laminotomy/hemilaminectomy with decompression), 63047 (lumbar laminectomy, facetectomy, and foraminotomy, single segment), and 63048 (additional vertebral segment decompression).

To define exposure groups, patients were required to have undergone preoperative MRI, identified using CPT codes 72148 (MRI lumbar spine without contrast), 72149 (MRI lumbar spine with contrast), and 72158 (MRI lumbar spine with and without contrast). MRI identification was based on CPT coding and may include variation in imaging scope (e.g., lumbar-specific vs. broader spinal imaging), which cannot be fully distinguished within the database.

In patients with multiple preoperative MRIs, the most recent MRI prior to surgery was used to define exposure timing. Patients were stratified into two cohorts based on the temporal relationship between MRI and surgical intervention. The early imaging cohort included patients who underwent MRI within 30 days prior to lumbar decompression (0-30 days). The delayed imaging cohort included patients who underwent MRI 31-90 days prior to surgery.

The index event for both cohorts was defined as the date of lumbar decompression surgery, corresponding to the first occurrence of CPT codes 63030, 63047, or 63048. MRI was required to occur prior to the index event within the defined time windows. Patients without a qualifying preoperative MRI within these timeframes were excluded.

To ensure appropriate temporal alignment, cohort definitions were constructed using TriNetX event relationships such that MRI occurred within the specified preoperative windows relative to the surgical procedure. Patients were excluded if an MRI occurred outside the defined intervals or after the index procedure.

Only the first qualifying surgical event per patient was included to avoid duplication. Patients with incomplete demographic data or age <18 years were excluded from the analysis. Surgical urgency (elective vs. emergent) is not reliably captured within the TriNetX database and, therefore, could not be included as a variable in the analysis.

Matching and covariate adjustment

To minimize confounding, propensity score matching (PSM) was performed within the TriNetX platform using a 1:1 nearest neighbor matching algorithm without replacement. Propensity scores were estimated via logistic regression incorporating predefined baseline covariates. A caliper width of 0.1 pooled standard deviations of the logit of the propensity score was applied, consistent with established methodology for minimizing selection bias in observational studies [7].

Covariates were selected a priori based on clinical relevance and included demographic variables (age at index, sex, race, and ethnicity) and comorbid conditions known to influence postoperative outcomes. Specifically, the following diagnoses were included using ICD-10 codes: diabetes mellitus (E08-E13), overweight and obesity (E66), chronic obstructive pulmonary disease (J44), chronic kidney disease (N18), major depressive disorder (F33), elevated blood pressure without hypertension diagnosis (R03.0), and sciatica (M54.3).

Matching was performed prior to outcome analysis to ensure comparability between the cohorts with respect to baseline characteristics. Covariate balance was assessed using standardized mean differences (SMDs), with an SMD <0.1 considered indicative of adequate balance. Following matching, all included covariates demonstrated adequate balance, indicating successful reduction of measurable confounding. The final matched cohorts included 37,245 patients in each group, and all subsequent analyses were conducted on the matched population.

Follow-up periods and outcomes

The index event for all patients was defined as the date of lumbar decompression surgery. Outcomes were assessed within a predefined follow-up period beginning one day after the index event and extending to 365 days postoperatively, consistent with the analytic framework used within TriNetX to exclude perioperative events occurring on the day of surgery.

The primary outcomes of interest included reoperation, postoperative infection, and postprocedural neurologic complications. Outcomes were defined using standardized coding systems.

Reoperation was defined as repeat lumbar decompression and related procedures, including wound exploration, revision, and surgical washout (incision and drainage), when identifiable within CPT coding constraints. Postoperative infection was defined using ICD-10 code T81.4 (infection following a procedure). Postprocedural neurologic complications were identified using ICD-10 code G97.1. For all outcomes, patients with a documented history of the outcome prior to the start of the follow-up period were excluded from the respective analyses to ensure that only incident postoperative events were captured.

Statistical analysis

All statistical analyses were performed within the TriNetX platform using built-in analytic tools. Following PSM, outcomes were compared between the cohorts using risk analysis, time-to-event analysis (Kaplan-Meier survival analysis), and number-of-instances analysis.

For categorical outcomes, risk ratios (RRs), odds ratios (ORs), and risk differences with corresponding 95% confidence intervals (CIs) were calculated. Statistical significance for risk differences was assessed using z-tests.

Time-to-event outcomes were evaluated using Kaplan-Meier survival analysis, with differences between cohorts assessed using the log-rank test. Hazard ratios with 95% CIs were calculated. For outcomes with multiple occurrences, the number-of-instances analysis was performed, reporting the mean, standard deviation, and median. Differences between the cohorts were assessed using independent-samples t-tests.

For all analyses, patients with the outcome prior to the start of the follow-up period were excluded. A two-sided p value of <0.05 was considered statistically significant.

## Results

Following PSM, 37,245 patients were included in each cohort. Baseline demographic and clinical characteristics were well balanced between the groups, with all SMDs <0.1 (Table 1). Outcome-specific denominators vary due to TriNetX cohort construction, in which only patients with available follow-up or outcome capture are included in each analysis.

**Table 1:** Baseline characteristics of propensity score-matched cohorts SD: standard deviation; SMD: standardized mean difference; COPD: chronic obstructive pulmonary disease; BP: blood pressure; MRI: magnetic resonance imaging

Characteristic	31-90 days MRI (n = 37,245)	<30 days MRI (n = 37,245)	SMD
Age, mean (SD)	57.5 (15.7)	57.5 (15.8)	0.001
Female, n (%)	16,648 (44.7%)	16,800 (45.1%)	0.008
Male, n (%)	20,596 (55.3%)	20,443 (54.9%)	0.008
White, n (%)	30,351 (81.5%)	30,193 (81.1%)	0.011
Black or African American, n (%)	3,353 (9.0%)	3,456 (9.3%)	0.010
Hispanic or Latino, n (%)	1,806 (4.8%)	1,803 (4.8%)	<0.001
Diabetes mellitus, n (%)	8,410 (22.6%)	8,618 (23.1%)	0.013
Overweight/obesity, n (%)	10,514 (28.2%)	10,547 (28.3%)	0.002
COPD, n (%)	2,978 (8.0%)	3,057 (8.2%)	0.008
Chronic kidney disease, n (%)	3,245 (8.7%)	3,323 (8.9%)	0.007
Major depressive disorder, n (%)	2,072 (5.6%)	2,188 (5.9%)	0.013
Elevated BP (no hypertension diagnosis), n (%)	2,089 (5.6%)	2,071 (5.6%)	0.002
Sciatica, n (%)	8,492 (22.8%)	8,447 (22.7%)	0.003

Reoperation

Reoperation occurred in 238 of 14,131 patients (1.7%) in the 31-90-day cohort and 313 of 17,451 patients (1.8%) in the <30-day cohort. There was no statistically significant difference between the groups (risk difference = -0.001, 95% CI = -0.004 to 0.002; p = 0.46; RR = 0.94, 95% CI = 0.80-1.11) (Table 2). Kaplan-Meier analysis demonstrated no significant difference in time to reoperation between the cohorts (log-rank p = 0.356), with survival probabilities of 98.16% in the 31-90-day cohort and 98.02% in the <30-day cohort at one year.

**Table 2:** Postoperative outcomes after lumbar decompression CI: confidence interval; MRI: magnetic resonance imaging

Outcome	31-90 days MRI (n = 37,245)	<30 days MRI (n = 37,245)	Risk difference (95% CI)	Risk ratio (95% CI)	p value
Reoperation	238/14,131 (1.7%)	313/17,451 (1.8%)	-0.001 (-0.004 to 0.002)	0.94 (0.80-1.11)	0.46
Postoperative infection	972/36,486 (2.7%)	1,197/36,331 (3.3%)	-0.006 (-0.009 to -0.004)	0.81 (0.74-0.88)	<0.001
Postprocedural neurologic complications	49/37,138 (0.1%)	55/37,153 (0.1%)	0.000 (-0.001 to 0.000)	0.89 (0.61-1.31)	0.56

Postoperative infection

**Figure 1:**
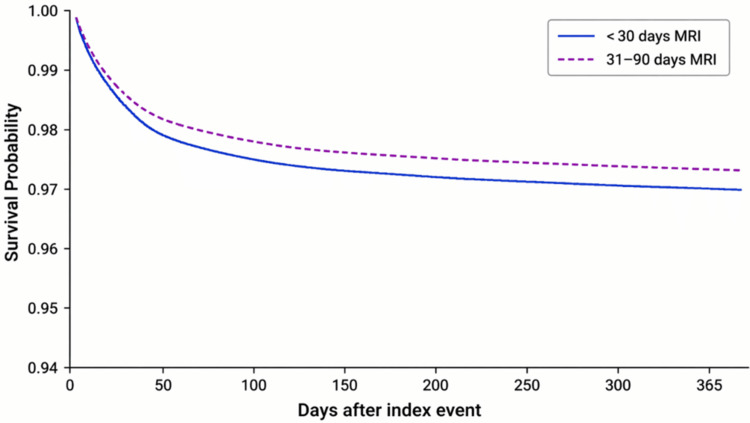
Kaplan-Meier curve for postoperative infection-free survival following lumbar decompression by timing of preoperative MRI Differences between cohorts were observed (log-rank p < 0.001) MRI: magnetic resonance imaging

Postoperative infection occurred in 972 of 36,486 patients (2.7%) in the 31-90-day cohort and 1,197 of 36,331 patients (3.3%) in the <30-day cohort. A difference between the groups was observed (risk difference = -0.006, 95% CI = -0.009 to -0.004; p < 0.001; RR = 0.81, 95% CI = 0.74-0.88) (Table 2). Kaplan-Meier analysis showed a difference in infection-free survival between the cohorts (log-rank p < 0.001), with survival probabilities of 97.24% in the 31-90-day cohort and 96.56% in the <30-day cohort at one year (Figure 1).

Postprocedural neurologic complications

Postprocedural neurologic complications occurred in 49 of 37,138 patients (0.1%) in the 31-90-day cohort and 55 of 37,153 patients (0.1%) in the <30-day cohort, with no statistically significant difference between the groups (risk difference = -0.000, 95% CI −0.001 to 0.000; p=0.56; RR 0.89, 95% CI 0.61-1.31) (Table 2). Kaplan-Meier analysis showed no significant difference in time to neurologic complications (log-rank p = 0.523), with survival probabilities of 99.86% and 99.84% in the 31-90 and <30-day cohorts, respectively.

## Discussion

In this large, propensity score-matched cohort of patients undergoing lumbar decompression, the timing of preoperative MRI within a 90-day interval was not associated with differences in reoperation rates or postprocedural neurologic complications. MRI performed 31-90 days prior to surgery was associated with lower observed postoperative infection rates compared with imaging obtained within 30 days of surgery. These findings should be interpreted as associative rather than causal, and likely reflect differences in underlying patient characteristics and care pathways rather than an independent effect of imaging timing.

Importantly, the observed association between shorter MRI-to-surgery intervals and higher infection risk should not be interpreted as causal. Rather, MRI timing likely serves as a surrogate marker for underlying clinical and system-level factors. Patients undergoing imaging closer to the time of surgery may represent a more acute or higher risk population requiring expedited intervention. Differences between elective and urgent surgical pathways are particularly relevant, as urgent or nonelective cases are associated with increased complication rates, including infection, in part due to limited opportunities for preoperative optimization [8]. Accordingly, the higher infection rates observed in the <30-day cohort are most consistent with confounding by indication, driven by differences in clinical acuity that are not fully captured in the dataset.

Conversely, patients with longer intervals between imaging and surgery may reflect more structured, elective care pathways, allowing for optimization of comorbid conditions and coordination of perioperative care. Processes such as prehabilitation and enhanced recovery protocols have been associated with improved outcomes and reduced complications in spine surgery populations [9,10]. As such, MRI timing likely reflects broader differences in healthcare delivery rather than exerting a direct influence on postoperative outcomes.

The absence of significant differences in reoperation and neurologic complication rates between the cohorts supports the premise that MRI timing does not substantially influence surgical planning or technical outcomes within a 90-day window. Prior literature has emphasized the importance of correlating imaging findings with clinical presentation rather than relying solely on imaging recency [11]. These findings suggest that, in clinically stable patients, preoperative MRI remains relevant without necessitating repeat imaging solely based on elapsed time.

Clinical implications

The findings of this study suggest that the timing of preoperative MRI within a 90-day interval is not associated with differences in major postoperative outcomes, including reoperation and neurologic complications, following lumbar decompression. These results support the clinical utility of MRI obtained up to three months prior to surgery in stable patients without interval changes in symptoms.

The observed association between MRI performed 31-90 days prior to surgery and lower postoperative infection rates likely reflects differences in patient selection and perioperative optimization rather than a causal effect of imaging timing. Patients undergoing expedited imaging and surgery may represent a higher risk population, underscoring the importance of targeted perioperative optimization in these cases.

From a practical standpoint, these findings suggest that repeat imaging based solely on elapsed time may not be necessary in the absence of clinical change. Avoiding unnecessary MRIs may reduce healthcare utilization and improve workflow efficiency. Further prospective studies are warranted to clarify the role of imaging timing within broader perioperative care processes.

Limitations

This study has several limitations inherent to its retrospective design and reliance on a large administrative database. Although PSM was used to balance measured covariates, residual confounding from unmeasured variables remains a major limitation. Key clinical factors, including surgical urgency (elective vs. emergent), symptom severity, neurologic deficits, and operative complexity, are not captured in the dataset and are likely the dominant sources of bias, particularly regarding postoperative infection risk.

Additionally, reliance on ICD-10 and CPT coding introduces the potential for misclassification bias. The definition of postoperative infection (T81.4) is broad and may include heterogeneous conditions, while neurologic complications (G97.1) may undercapture clinically meaningful deficits. Variability in coding practices across institutions may further affect the accuracy of cohort identification and outcome assessment.

The database also lacks granular intraoperative and perioperative details, including operative duration, surgical technique, antibiotic prophylaxis, and adherence to infection prevention protocols, all of which may influence outcomes. Furthermore, MRI identification is based on CPT coding and does not allow for differentiation between lumbar-specific and whole-spine imaging.

Outcome-specific denominators vary due to cohort construction within the TriNetX platform, where only patients with available follow-up for each outcome are included, potentially contributing to differences in sample size across analyses.

Selection bias is also likely present, as patients undergoing MRI closer to surgery may represent a more acute population requiring expedited intervention. Although matching was performed, differences in care pathways and clinical urgency cannot be fully accounted for. Finally, while the large, multi-institutional dataset enhances generalizability, it may introduce heterogeneity in patient populations and surgical practices that could influence outcomes.

## Conclusions

In this large propensity score-matched analysis, the timing of preoperative MRI within 90 days before lumbar decompression was not associated with differences in reoperation or postprocedural neurologic complications. MRI performed 31-90 days before surgery was associated with lower observed rates of postoperative infection compared to imaging obtained within 30 days of surgery. These findings should be interpreted as associative rather than causal and likely reflect underlying differences in patient acuity, surgical urgency, and care pathways rather than an independent effect of imaging timing. Further prospective studies are warranted to better define the relationship between imaging timing and postoperative outcomes within the context of perioperative care.

## References

[1] Deyo RA, Mirza SK, Martin BI (2006). Back pain prevalence and visit rates: estimates from U.S. national surveys, 2002. Spine (Phila Pa 1976).

[2] Modic MT, Ross JS (2023). Lumbar Degenerative Disk Disease. Radiology.

[3] Carragee EJ, Alamin TF, Miller JL, Carragee JM (2005). Discographic, MRI and psychosocial determinants of low back pain disability and remission: a prospective study in subjects with benign persistent back pain. Spine J.

[4] Jarvik JG, Deyo RA (2002). Diagnostic evaluation of low back pain with emphasis on imaging. Ann Intern Med.

[5] Schoenfeld AJ, Carey PA, Cleveland AW 3rd, Bader JO, Bono CM (2013). Patient factors, comorbidities, and surgical characteristics that increase mortality and complication risk after spinal arthrodesis: a prognostic study based on 5,887 patients. Spine J.

[6] (2026). TriNetX global collaborative network. https://live.trinetx.com.

[7] Austin PC (2011). An introduction to propensity score methods for reducing the effects of confounding in observational studies. Multivariate Behav Res.

[8] Pugely AJ, Martin CT, Gao Y, Mendoza-Lattes S (2014). Causes and risk factors for 30-day unplanned readmissions after lumbar spine surgery. Spine (Phila Pa 1976).

[9] Ljungqvist O, Scott M, Fearon KC (2017). Enhanced recovery after surgery: a review. JAMA Surg.

[10] Debono B, Sabatier P, Garnault V, Hamel O, Tessier P (2016). Enhanced recovery after surgery (ERAS) program in spine surgery. Orthop Traumatol Surg Res.

[11] Boden SD, Davis DO, Dina TS, Patronas NJ, Wiesel SW (1990). Abnormal magnetic-resonance scans of the lumbar spine in asymptomatic subjects. A prospective investigation. J Bone Joint Surg Am.

